# Diversity and Interactions of Wood-Inhabiting Fungi and Beetles after Deadwood Enrichment

**DOI:** 10.1371/journal.pone.0143566

**Published:** 2015-11-24

**Authors:** Andreas Floren, Dirk Krüger, Tobias Müller, Marcus Dittrich, Renate Rudloff, Björn Hoppe, Karl Eduard Linsenmair

**Affiliations:** 1 Department of Animal Ecology and Tropical Biology, Biocenter, University of Würzburg, Am Hubland, D-97074, Würzburg, Germany; 2 Department of Soil Ecology, UFZ—Helmholtz Centre for Environmental Research, Theodor-Lieser-Straße 4, D-06120, Halle (Saale), Germany; 3 Department of Bioinformatics, Biocenter, University of Würzburg, Am Hubland, D-97074, Würzburg, Germany; Georg-August-University Goettingen, GERMANY

## Abstract

Freshly cut beech deadwood was enriched in the canopy and on the ground in three cultural landscapes in Germany (Swabian Alb, Hainich-Dün, Schorfheide-Chorin) in order to analyse the diversity, distribution and interaction of wood-inhabiting fungi and beetles. After two years of wood decay 83 MOTUs (Molecular Operational Taxonomic Units) from 28 wood samples were identified. Flight Interception Traps (FITs) installed adjacent to the deadwood enrichments captured 29.465 beetles which were sorted to 566 species. Geographical ‘region’ was the main factor determining both beetle and fungal assemblages. The proportions of species occurring in all regions were low. Statistic models suggest that assemblages of both taxa differed between stratum and management praxis but their strength varied among regions. Fungal assemblages in Hainich-Dün, for which the data was most comprehensive, discriminated unmanaged from extensively managed and age-class forests (even-aged timber management) while canopy communities differed not from those near the ground. In contrast, the beetle assemblages at the same sites showed the opposite pattern. We pursued an approach in the search for fungus-beetle associations by computing cross correlations and visualize significant links in a network graph. These correlations can be used to formulate hypotheses on mutualistic relationships for example in respect to beetles acting as vectors of fungal spores.

## Introduction

Deadwood is an important habitat and structural component in forest ecosystems developing often into biodiversity hotspots. It provides shelter and nutrition to various organisms, primarily fungi and saproxylic insects [1–3]. Wood-inhabiting fungi are key players in forest ecosystems due to their ability of decomposing wood, recycling nutrients and initiating a successional dynamic for saproxylic arthropods [4,5]. Many studies have focused on the effects of resource availability for fungal and beetle communities [6,7] and how communities are affected by anthropogenic disturbance [8–10]. The amount and quality of deadwood have been shown to be crucial for saproxylic organisms [11–13]. As deadwood availability is greatly influenced by forest management type the interrelation of these factors and their impacts on biodiversity and ecosystem functioning has become a major topic in research [14,15].

Recent findings indicate that wood-inhabiting fungi can discriminate forest types [16,17]. This is remarkable because spores are usually assumed to be dispersed easily in forests although studies suggest that some wood-inhabiting fungi might actually be dispersal limited [4,18]. This raises questions about the mechanisms determining the spatial distribution of fungi. Recent research hints towards the role of arthropods as vectors for fungal spores [19]. For example, investigations indicated that bark beetles facilitate the establishment of wood-inhabiting fungi [20,21]. Although the role of zoochory has been considered low besides for mutualistic relationships [22,23,24] more and more studies indicate that vectoring insects can influence fungal community composition [19,20]. However, examples of effective insect vectors refer mostly to economically important associations like those between bark beetles (Scolytidae) or plant wasps (Symphyta) and their fungal associates [25,26]. The role of saproxylic arthropods in the process of wood decay is more controversially discussed [27]. Saproxylic arthropods are mainly considered to mechanically reduce wood to small pieces extending the surface area and allowing microorganisms to decompose woody material more easily [28]. Fungal spore transport might be of greater importance in this context by influencing fungal community composition and thereby also the effectivity of wood decomposition [5,19,29]. Apart from the importance the arthropods play for the colonisation of wood-decaying fungi priority effects during the colonisation of fungi might also lead to the establishment of differently structured fungi communities thus influencing the succession of saproxylic arthropods [30]. Other fungus-beetle relationships could be even more sophisticated. For example, fungal volatiles may also attract beetle species influencing beetle community composition [31]. Such relationships are little investigated topics in research.

Our research aims to narrow this gap of knowledge by analysing how wood-inhabiting fungi and beetle assemblages respond to local habitat conditions and to what extent both groups show similar patterns in vertical stratification (ground–canopy) as suggested in earlier investigations [32,33]. We also test for the effects of forest management on assemblage composition and ask how sensitive taxa respond to changing environmental conditions. Finally, we use the abundance data for a cross-correlation in order to search for possible indications of fungus-beetle associations. In this context, our study can be only seen as a precursor step for a subsequent detailed screening for species interactions.

## Material and Methods

### Study sites

Research was performed in three distant regions of Germany that were established within the “Biodiversity Exploratories” project [34]. These were the UNESCO Biosphere Reserve Swabian Alb (Alb) in South-West Germany, the Hainich-Dün exploratory containing the UNESCO World Heritage at Hainich Mountains and adjacent areas such as the Dün Mountains in Central Germany (Hainich) and the UNESCO Biosphere Reserve Schorfheide-Chorin (Chorin) in North-East Germany (S1 Fig). Distance between each region from South to North is approximately 300 km. All regions are further characterized by different representative soil types (Schwabian Alb: Cambisol/Leptosol; Hainch-Dün: Luvisol/Stagnosol; Schorfheide-Chorin: Cambisol). In each region several beech forests managed with differing intensities were selected. Three types of land use intensity were distinguished, 1) forests left unmanaged for 20–70 years (unm), 2) forests from which only individual trees or small groups are harvested resulting in an uneven age-structure (ext) without further invasive management activities in order to restore structurally more complex beech stands and 3) age-class forests (acf) which are characterised by uniform tree species composition, forest structure and site conditions. The latter forests are managed for efficient timber extraction in 60 to 100 years intervals. Altitudes vary between 600–860 m a.s.l. in the Alb, 290–550 m in Hainich and 3–140 m in Chorin. Annual mean precipitation is between 700–1000 mm in the Alb and 500–800mm in the other areas while the range of mean temperature lies between 6–8.5°C. Landscapes consist of a small-scale mosaic of grasslands and forests with European beech (*Fagus sylvatica*) as economically most important deciduous tree species. Within these landscapes a total of 16 forest plots were selected (S2 Fig) which were separated from each other by several kilometres of open land or other forests.

### Deadwood enrichment

Deadwood was artificially enriched in the canopy and near the ground of a single study tree in each forest plot in 2009 (S2 Fig). All wood was taken from a single freshly felled forest stand of each of the three areas to keep the experimental set up, like existing endophytic fungi, as comparable as possible. Sterilisation of the freshly cut deadwood was not possible due to the amounts used in the experiment. At the time of this investigation the deadwood had been left decomposing already for two years. The experimental set-up was based on the assumption that amounts of deadwood are too low to sustain functionally diverse communities of saproxylic arthropods. It has been extensively described in Floren et al. [32] so that we provide only basic information here. The enrichment experiment used three log sizes (diameters 1–5 cm, 6–10 cm, 11–20 cm). In the canopy, bundles of dead-wood were fixed at the trunk with wire mesh and steel rope at 22 m, 18 m and 16 m height (smallest size on top and largest size below). Each size of deadwood contained about 0.2 m^3^ so that the total amount was 1.2 m^3^ per tree. In addition, three stacks of cut wood (each 1 m^3^) of the same three sizes were piled up on the ground beneath the study trees. Such large quantities were used to overcome resource limitation. Logs were placed on a wooden underlay to delay soil fungi from colonising. FITs were installed next to each deadwood bundle to continuously sample arthropods in a jar mounted at the bottom of each FIT. A 1.5% cuprum-sulphate solution was used as killing and conserving liquid. All traps were installed in February 2011 and emptied monthly from April to the end of September. Beetles from the two individual trees per plot were pooled for the analysis.

### Collection of beetles and deadwood drill samples for identification of fungi

The following recordings aim at documenting the importance of colonizing arthropods and wood-inhabiting fungi during the decomposition of deadwood. Beetles were collected close to the deadwood that was artificially enriched in the canopy and near the ground of a single study tree in each forest plot in 2009 (S2 Fig). At the time of this investigation the deadwood had been left decomposing already for two years. Wood-inhabiting fungi were detected by molecular methods from drill samples obtained from the same logs that follow a procedure which is described in detail in [35]. Briefly, in 2011 wood chips from the deadwood logs of the experiment were sampled using an electronic drill machine. One drill core each was sampled from the large sized wood stack. To avoid contamination between samples, the wood auger was flamed and wiped with ethanol between each core. The wood samples were kept on dry ice and later stored at -80°C upon return to the lab.

### Isolation of fungal DNA

The total community DNA from 0.5g of each wood sample was isolated using a modified CTAB-protocol [36] as described in Hoppe et al. [37]. Briefly, 900 μl of CTAB was added to the sample and nucleic acids were separated from proteins and cell debris by adding 500μl of 24:1 chloroform: isoamyl alcohol (Carl Roth, Karlsruhe, Germany) followed by another chloroform step (Carl Roth). DNA was precipitated and washed twice with 99% ethanol (Merck, Darmstadt, Germany). Dried DNA pellets were dissolved in 100 μl molecular grade water (AppliChem, Darmstadt, Germany).

### PCR, cloning and sequencing

The primer pair ITS1-F [38] and ITS4 [39] was used to amplify the ITS region of fungal rDNA. The PCR of each DNA extract was done separately in duplicates in a 30 μl-reaction mixture containing 15 μl GoTaq Green Master Mix 2x (Promega, Mannheim, Germany), 10 μM of each primer and approximately 20ng template DNA. PCR was performed with an initial denaturation step at 95°C for 5 min followed by 35 cycles at 95°C for 60 s, 55°C for 60 s and 72°C for 75 s. Elongation was completed with a final step of 72°C for 7 min. After quality check of PCR products on a 1.5% agarose gel the duplicates were pooled for one cloning reaction followed by a purification using E.Z.N.A.® Cycle-Pure Kit (Omega Bio-Tek, Inc., Norcross, GA, USA).

Cloning was done with pGEM-T Vector System (Promega GmbH, Mannheim, Germany) with competent *Escherichia coli* JM109 according to the manufacturer’s instructions. Approximately 32 clones per clone library were PCR screened for the insert by re-amplification using primers M13F and M13R and the following PCR-conditions: 95°C for 5 min, 32 cycles of 95°C for 40 s, 54°C for 30 s and 72°C for 60 s and a final elongation step of 72°C for 10 min. Amplicons were checked on 1% agarose gels under UV light. Clones with insert were purified with ExoSAP-IT (USB Corporation, Cleveland, Ohio, USA) and then used in cycle sequencing with the M13 primers as sequencing primers using Big Dye Terminator Cycle Sequencing Reaction Kit v.3.1 (Applied Biosystems, Foster City, CA, USA). After an ethanol precipitation sequencing was done on an ABI 3730xl DNA Analyzer (Applied Biosystems, Foster City, CA, USA), using both M13F and M13R in parallel.

### Sequence analyses

Editing and trimming of primer residues were done using Sequencer 4.10. (Genecodes, Ann Arbor, MI, USA). Contigs for exporting as consensus sequence were built from the complementary forward and reverse sequences. Identification of all exported sequences was done by BLASTn query against the DDBJ nucleotide sequence database using standard settings but excluding the environmental division and targeting the plant division that includes all fungi in that database. The ten best hits were recorded; if discrepancies were found or non-fungal hits included, 50 were recorded. The sequences were immediately given the exact name of the best hit with at least an available genus name (if available) and without any conflict between the top hits; otherwise a next hit with more precise name was used. Sequences were checked for chimeras using UCHIME [40] in USEARCH v. 6.0 [41] in de-novo mode. Sequences unalignable in ClustalX2 [42] and at the same time having unusual ITS2 secondary structure [43], or not covering primer-to-primer, or stemming from only one good sequencing reaction instead of from both forward and reverse sequencing, or not identified as fungal, or flagged as chimeric were excluded. Ultimately we normalized to 26 sequences per sample by random removal of excess sequences.

Building of MOTUs and dereplication were then performed using the BLASTclust module of the NCBI-BLAST package ported at http://toolkit.tuebingen.mpg.de/blastclust specifying as coverage 80% and as identity threshold 97% for general fungal ITS. MOTUs were sequentially numbered by descending sequence membership, appended by the consensus genus name from all individual sequences in the MOTU. MOTU reference sequences are available from the International Nucleotide Sequence Database Collaboration portals under DDBJ accessions LC015665—LC015745 upon publication of this article.

### Statistics

Alpha diversity was calculated as exponential Shannon diversity which calculates an ‘effective number of species’ [44]. Rarefaction statistics (sample- and individual based) were computed to allow direct comparison of diversity between unevenly sampled species pools. Similarity of beetle assemblages was analysed by canonical correspondence analysis (CCA). Significance of the factors ‘area’, ‘vertical stratum’ and ‘forest management’ were assessed by an ANOVA like permutation test implemented in the vegan package (model formulation: cca(species.data ~Management + Stratum + Condition (area)).

In order to find associations between beetle species and fungal MOTUs we performed a co-inertia analysis as well as a correlation analysis [45]. The two ecological data sets (beetles and fungi) produced two representations of the sites in two hyperspaces. Separate analyses identified axes maximising inertia in each hyperspace. Co-inertia analysis aims to find a couple of co-inertia axes on which the sites are projected. The co-inertia analysis calculates an RV-coefficient which is the ratio of the total co-inertia to the square root of the product of the squared total inertias of the separate analyses. RV is a multivariate generalization of the Pearson correlation coefficient and varies between 0 and 1. Its strength is tested by a permutation test. All statistical analyses were performed in R (R Development Core Team 2011) using the packages ‘vegan’ [46] and ‘ade4’ [47].

For the prediction of the interaction graph we used only species of beetles and fungal MOTUs that were collected from at least 5 plots for a cross-correlation (Spearman rank correlation). Few phytophagous species passed this filter (see below). As they do not negatively influence the results of the cross-correlations but as they might play a passive role in transmitting of fungal spores they were not excluded from this analysis.

Statistical results were based on 10.000 permutations (sampling within rows). We also calculated the F-statistics for comparison. Significance values were adjusted according to Benjamini-Hochberg. Displayed are all interactions above a level of corrected significance of 0.1. The significant interactions were plotted as a graph indicating positive and negative correlations between fungi and beetle pairs.

## Results

### Diversity of fungi and beetles

We retained a total of 726 sequences from the 28 wood samples of which a 97% divergence cutoff resulted in 81 fungal MOTUs (Alb = 31, Chorin = 35, Hainich = 42). S3 Fig contains an icicle chart with a matrix-like overview of the community composition along with the reference sequence database accession numbers and all the sequences obtained in FASTA format.

**Fig 1:**
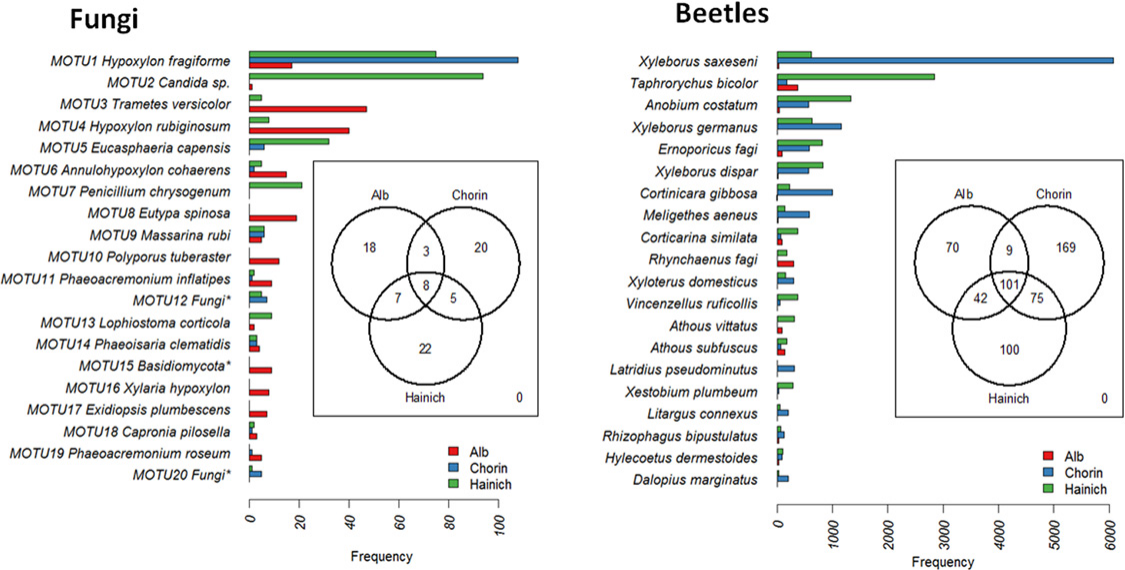
The 20 most abundant MOTUs (left) and species of beetles (right) respectively collected from all regions. Embedded Venn diagrams show the low proportion of taxa occurring in all regions. * = lower taxonomic rank could not be determined for the MOTUs labeled as Fungi and Basidiomycota

**Fig 2:**
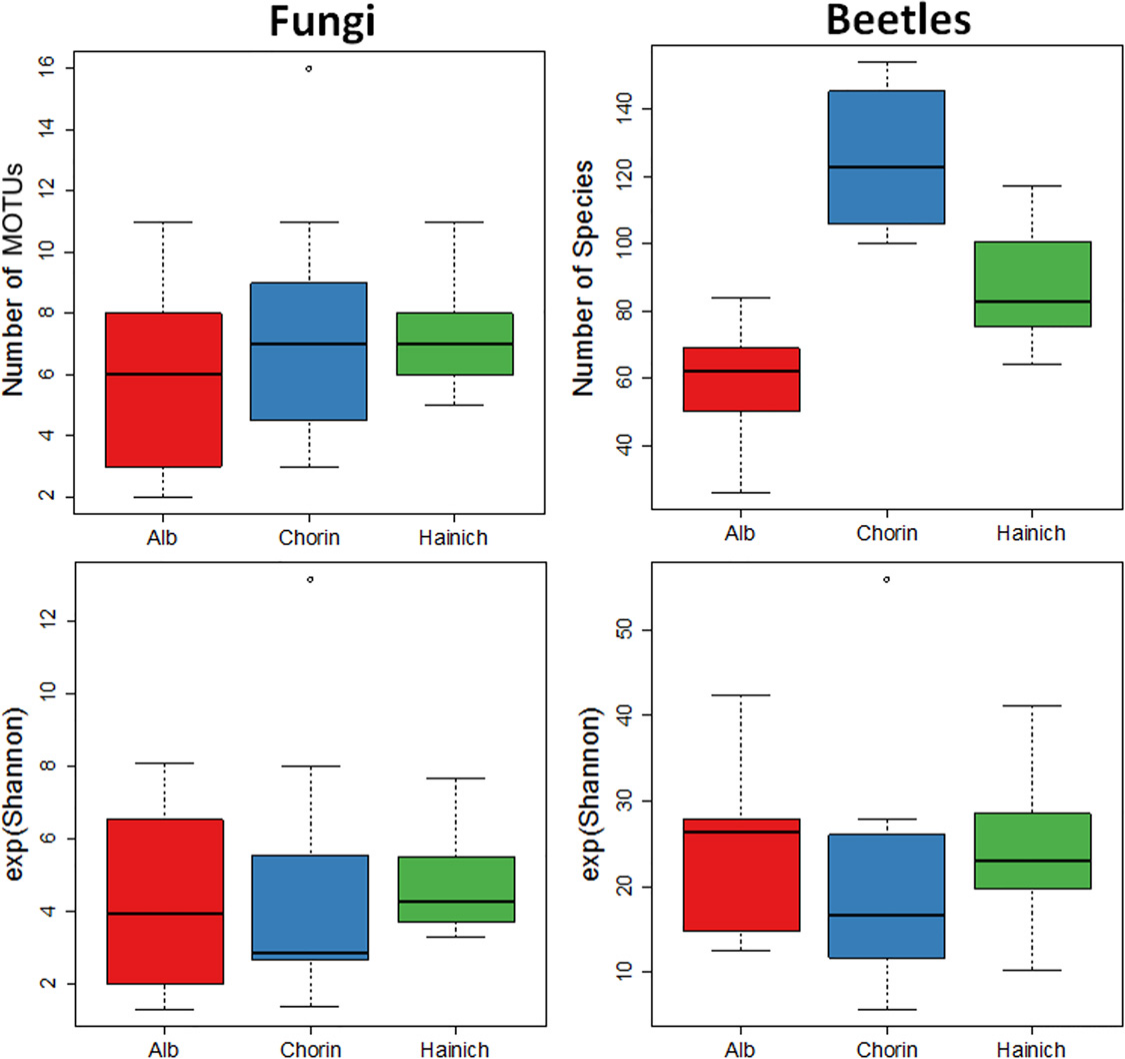
Boxplots visualising species richness and exponential Shannon diversity of wood-inhabiting fungi (left) and beetles (right). Mean numbers of beetle species differed significantly between regions (Kruskal p<0.001) while Shannon diversity showed no significant differences due to high unevenness. For wood-inhabiting fungi the regional effects were statistically not significant.

In total 44 MOTUs were isolated from the canopy deadwood (13 MOTUS or 30% sampled as singletons) and 60 from the ground stacks (22 MOTUS or 37% singletons). Ascomycota dominated (63 MOTUs, 77%) followed by Basidomycota (15 MOTUs, 18%). *Hypoxylon fragiforme* (*Xylariaceae*) and *Candida* sp. (Saccharomycetales) were most common (Fig 1 and S3 Fig). The 29.465 beetles were sorted to 566 species of which 356 species (64.5% of the total) were found in 16.800 specimens (57%), while 455 species (80.4%) were caught in 12.665 individuals (43%) near the ground. Richness decreased following a North-South gradient: Chorin (354 species, 14.903 specimens), Hainich (318 species, 12.335 specimens), Alb (222 species, 2.227 specimens). For both fungi and beetles we observed a small overlap across regions namely eight fungal MOTUs (9.9%) and 101 beetle species (17.8%) (Fig 1). Twelve percent of all species were exclusively collected in the Alb, 18% in the Hainch region and 30% in Chorin. Similarity in species composition decreased with increasing distance between areas for fungi and beetles as also indicated by the frequency distribution of the most common species. Low similarity in species composition was one reason why sampling was not representative (S4 Fig). In contrast to wood-inhabiting fungi, the mean number of beetles per tree differed between areas (Kruskal test p<0.001) with highest richness recorded from Chorin and lowest richness from the Alb (Fig 2 and compare S3 Fig). Species diversity differed not significantly between areas neither for fungi nor for beetles.

### Effects of vertical stratification and forest management

We computed a cca model to assess the effect of stratum and forest management on the complete data of beetles and fungi. The model revealed a significant region effect for both fungi and beetle assemblages (p<0.01). Consequently, we conditioned on region in subsequent analyses. We also reduced the influence of rare species by computing a similar model on a reduced data set with species found in at least three samples [48]. The filtered data sets included 20 fungal MOTUs and 255 species of beetles. The influence of management and stratum on fungi assemblages revealed borderline associations for stratum (p = 0.086) and management (p = 0.067). Beetles showed only a significant stratum association (p<0.01) but no management effect (p = 0.280).

**Fig 3:**
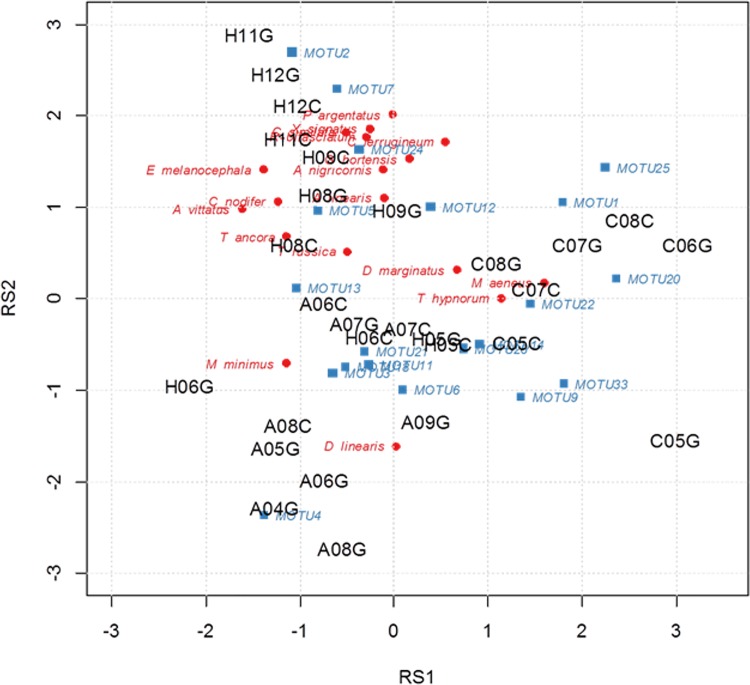
Co-inertia analysis based on species found at least in three samples. The data sets of beetles and fungi produced two representations of the sites in two hyperspaces. Analysis aims at maximising inertia in each hyperspace. Only extreme species are plotted. A = Swabian Alb (AEW plots), H = Hainich-Dün (HEW plots), C = Schorfheide-Chorin (SEW plots). The letter at the end of the study plot abbreviation indicates whether sample was collected from the canopy (C) or the ground (G).

We measured the strength of the relationship between fungi and beetles by performing a co-inertia analysis which resulted in a clear separation of all areas (Fig 3) suggesting the existence of associations between fungi and beetles (RV test, 5,999 permutations, p<0.001). However, after computing the model for each single area no significant fungus-beetle association was found (RV test, Observation: 0.575, 5,999 replicates, p = 0.222). This indicates that either the data for each area were too small to discover species-specific associations or that the statistical power was too low. Fig 3 shows also that the assemblages in the Alb and Chorin exhibit only a vague separation between management types and stratum. In contrast, the unmanaged National Park forests in the Hainich (HEW plots 11, 12) are clustered together and group close to the extensively managed forests while both separate from the age-class forests which are in fact more similar to assemblages in the Alb.

### Detailed analyses of the Hainich-Dün exploratory data

**Fig 4:**
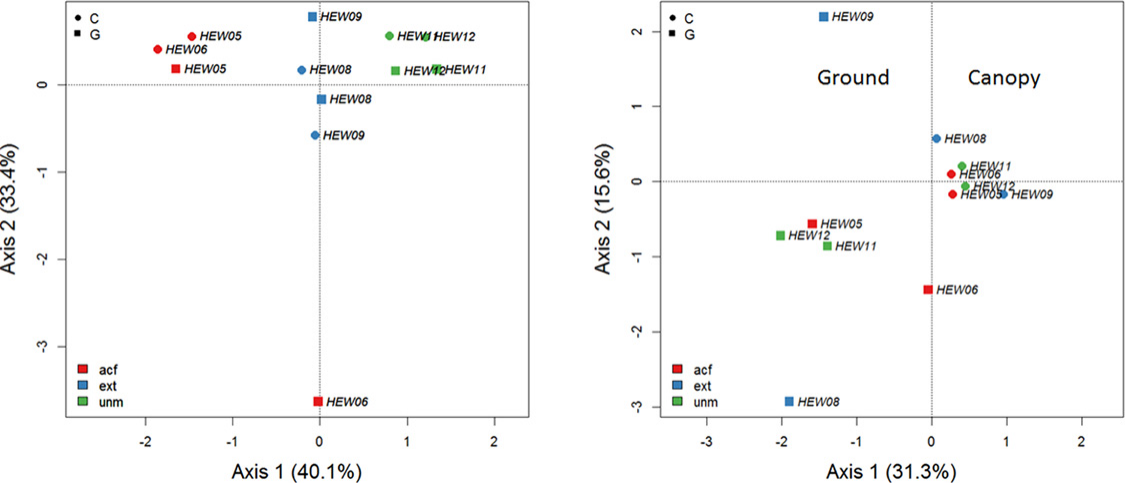
Ordination plots on Hainich data of all species collected at least two times (left: fungal assemblages, right: beetle assemblages). On the first axis wood-inhabiting fungi respond to forest management while FIT-sampled beetles are characterised by the factor stratum without showing a management effect; acf = age-class forest, ext = extensively managed forest, unm = unmanaged forest; C = canopy, G = ground.

The following analysis was restricted to the Hainich dataset which most comprehensively represented all management types. Further reasons to focus on the Hainich data were: we only observed a weakly significant stratum effect for beetles in the Alb (p<0.05) and there was no significant response for either fungi or beetles in Chorin. The reduced and filtered Hainich data consisted of the most common 7 fungal MOTUs and 131 species of beetles. Model results suggest that wood-inhabiting fungi discriminate between management types (p<0.001) but not between stratum (formula = species ~ management + stratum). In contrast, beetles showed a significant stratum effect (p<0.01) but did not respond to management (p = 0.46). Most beetle species were zoophages (48 species), followed by mycetophages (36 species), xylophages (23 species), phytophages (17 species) and saprophages (6 species). How clearly fungi and beetle assemblages separate becomes obvious by ordination (Fig 4). The clear separation of wood-inhabiting fungi (left part of Fig 4) between management types is disturbed by just a single outlier (HEW06).

### Interaction of wood-fungi and beetles

**Fig 5:**
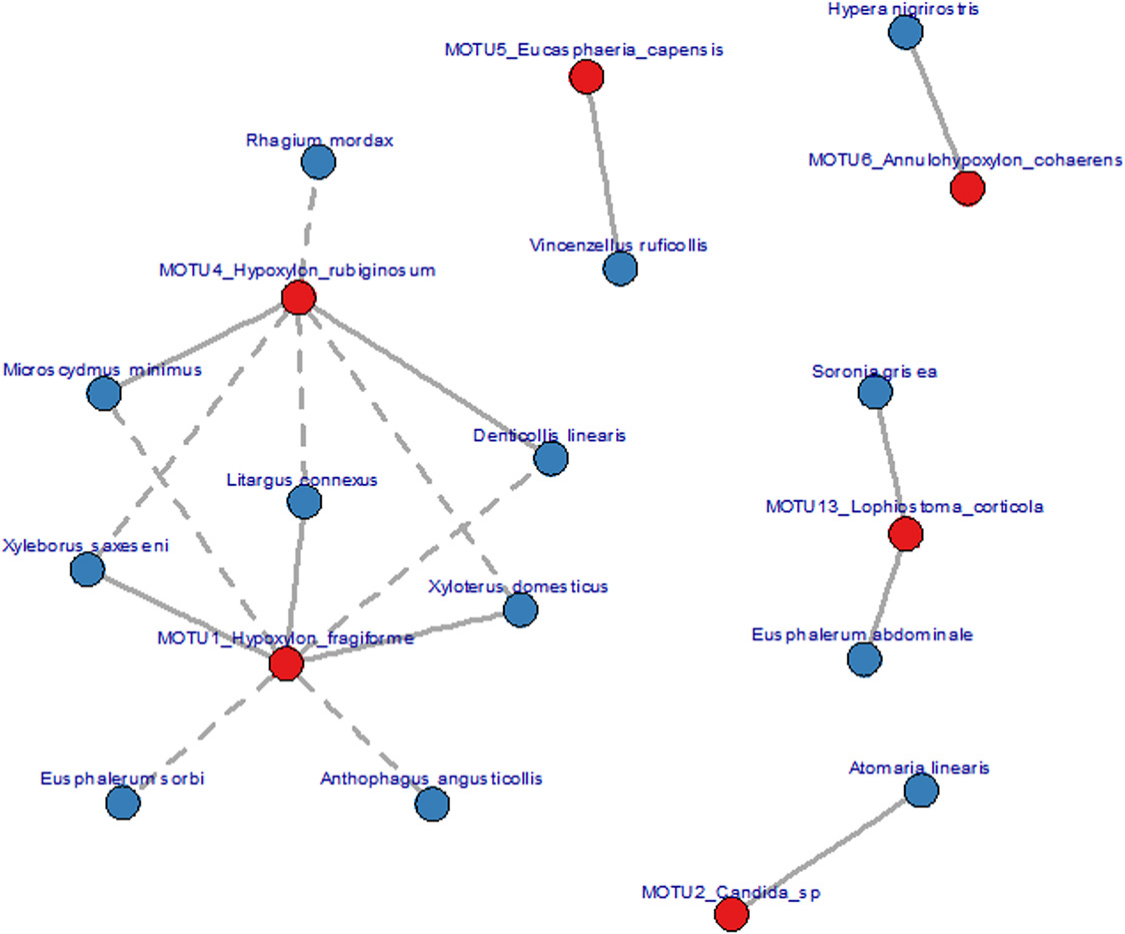
Graph visualising significant Spearman correlations (based on 10.000 random permutations) between fungi and beetles in the Hainich data. Solid lines represent positive correlations while dotted lines denote negative correlations. Fungi MOTUs are shown in red, beetle species in blue.

In search for single fungus-beetle associations we performed a cross-correlation analysis between fungi and beetle species (Fig 5). Using only species occurring in at least five plots there remained 152 beetle species and 11 fungi MOTUs resulting in a total of 1.672 Spearman rank correlations between beetle-fungus pairs. After adjustment for multiple comparisons the exploratory significance cut-off was set at 0.1 resulting in 18 significant beetle-fungi interactions which are graphically represented in Fig 5 (see also Table 1). Beetles of most fungus-beetle associations identified were saproxylics or mycetophages. Only three species were phytophages namely *Eusphalerium sp*. (Staphylinidae) and *Hypera* (Curculionidae). The dominant fungal genera was *Hypoxylon* (*Xylariaceae*) represented by the two species *H*. *fragiforme* and *H*. *rubiginosum*. *Annulohypoxylon* is a closely related genus only segregated from *Hypoxylon* in 2005.

**Table 1:** Significant associations between wood-inhabiting fungi and beetles in the Hainich region.

Fungi	Beetle	Correlation	p-value
MOTU4 *Hypoxylon rubiginosum*	*Microscydmus minimus*	0.7977	1.00E-04
MOTU2 *Candida sp*.	*Atomaria linearis*	0.7742	1.00E-04
MOTU4 *Hypoxylon rubiginosum*	*Xyleborus saxeseni*	-0.7688	2.00E-04
MOTU1 *Hypoxylon fragiforme*	*Denticollis linearis*	-0.7687	1.00E-04
MOTU1 *Hypoxylon fragiforme*	*Xyleborus saxeseni*	0.7651	1.00E-04
MOTU1 *Hypoxylon fragiforme*	*Microscydmus minimus*	-0.7348	1.00E-04
MOTU1 *Hypoxylon fragiforme*	*Litargus connexus*	0.7239	1.00E-04
MOTU4 *Hypoxylon rubiginosum*	*Denticollis linearis*	0.7096	3.00E-04
MOTU13 *Lophiostoma corticola*	*Soronia grisea*	0.7063	3.00E-04
MOTU13 *Lophiostoma corticola*	*Eusphalerum bdominale*	0.7054	6.00E-04
MOTU4 *Hypoxylon rubiginosum*	*Litargus connexus*	-0.6678	5.00E-04
MOTU5 *Eucasphaeria capensis*	*Vincenzellus ruficollis*	0.6648	4.00E-04
MOTU6 *Annulohypoxylon cohaerens*	*Hypera nigrirostris*	0.6374	0.0019

## Discussion

### Characterisation of fungal and beetle assemblages

Analysing the diversity and distribution of fungi and beetles attracted to beech deadwood revealed large regional differences between three distant German forest sites (S1 Fig). Similar results had been published by Giordano et al. [49], who investigated fungal assemblages on the bark beetle *Ips typographus* at different locations. Beetle richness was lowest in the mountainous Swabian Alb where climatic conditions, soil, vegetation and other factors are less favourable compared to the lowlands [50].

In the Alb, only 8% of the almost 30.000 beetle individuals and 39% of the total 566 species were collected. The Hainich region on the other hand harboured 56% of all species and Chorin 63% in similar numbers of beetle individuals. For wood-inhabiting fungi the regional effects were statistically not significant (Fig 2). This is in line with a broad investigation on 117 deadwood logs of *Fagus sylvatica*, where it has been shown, that geographical distances are not impacting fungal community composition [51]. As in most investigations species richness was not sampled representatively for neither taxa (S3 Fig) due to high proportions of singletons and rare species [8,32]. This indicates not only the large number of samples required to representatively collecting local assemblages but that large areas are needed to protect this diversity [52]. In contrast to the fungal species that were directly identified from the deadwood the FITs collect flying arthropods in a rather unspecific way. However, due to our experimental set up (S2 Fig) the majority of all beetles (species and specimens) were saproxylics that were attracted to beech wood [11].

Saproxylic beetles and wood-inhabiting fungi are considered indicators of wood decay allowing for the documentation and analyses of changes in the diversity and distribution in different forests. High abundance of ascomycetes (*Xylariaceae*) and unusually low numbers of basidiomycetes suggest that wood decomposition was even after two years in its initial phase. Similar results had been found by Strid et al. [19]. It is also in line with findings of Hoppe et al. [35] who reported a significant higher diversity of Ascomycota on beech deadwood logs of different decay stages while Basidiomycota ITS was detected in higher abundance across all decay classes. Slow decay process is furthermore suggested by high abundance of the early colonizing fungus *Hypoxylon fragiforme* (*Xylariaceae*) in the beech logs, see also [53], while *H*. *rubiginosum*, which typically colonises older barkless beech wood, was detected in much lower abundance. In addition to these findings, an early stage of wood decay is also indicated by the many saproxylic beetles that are indicative for fresh deadwood [54,55]. From the ten most abundant species of beetles which accounted for two third of all beetle individuals only the xylophagous species *Anobium costatum* (Latridiidae) has been classified to preferentially use old deadwood. Therefore, the data represents only the early colonisation of beech deadwood. As ascomycetes are considered to provide a key role for the creation of new habitats in later stages of wood decomposition one should correspondingly expect higher richness and a larger complexity of interactions between species [52]. However, due to the lack of long-term investigations respective knowledge is rare.

Many typical wood-rotting basidiomycetes quickly colonize wood in a very aggressive manner (e.g. *Fomes fomentarius*, *Trametes versicolor*; [35]) and have been shown to reside as endophytes in living trees [56]. However, these were not detected. Furthermore, basidiomycetes are not known to occur in small diameter deadwood like those used in the experimental set-up probably because they fail to fruit there. *Pro parte*, the yet not well distinguishable ground and canopy fungal diversity might be due to remnant effects, e.g. part of those fungi has been in the wood at the setup of the experiment already. Future investigations must provide clarification.

### The importance of stratum and management

Vertical stratification (canopy vs. ground) was found to affect the composition of the beetle assemblages in our study. This has only recently been demonstrated convincingly for beetle assemblages in temperate forests [32,57] since clear stratification was assumed to occur mainly in tropical rain forests. With respect to the wood-inhabiting fungi much less is known about the factors influencing their distribution. The stratum effect for the fungi was weak and not confirmed for each region. It has been shown recently, that even across a 600km latitude gradient, no significant variation in deadwood fungal community composition occurred [51]. Therefore, substrate quality and priority effects might rather serve as predictors for colonisation patterns [35,58]. With progressing wood decay and decreasing abundances of the *Xylariaceae* we expect an increasing impact of the stratum effect on fungal community composition.

Our results provide further evidence that forest management may alter fungal diversity and community composition [10,11,53]. Depending on deadwood, fungal richness is expected to increase in more natural forests where deadwood is available in larger amounts. Forest fragmentation is another factor determining community richness and structure [5]. It was surprising how finely fungal communities can distinguish near-to-nature logging schemes as this became particularly obvious in the Hainich where the management gradient was most pronounced. Here, even the extensively managed selection forests were clearly separated from unmanaged National Park plots. The degree of clarity of these findings demand further studies. Particularly interesting is the question whether alterations on the community level correspond with functional shifts [14] which might influence for example rates of wood decomposition [5]. As changes in communities are accompanied by alterations in forest structure that also affect the microclimatic conditions [33] community level adaptations should be expected.

Despite good indications that beetle assemblages react sensitively to forest management our data do not provide respective evidence to this. Since a management effect has been documented earlier on a more comprehensive data set for saproxylic beetles [32] the lack of proof is probably a consequence of the limited data used in this investigation.

### Beetles-fungi interaction

Fungi are the key organisms in the process of wood decay. In contrast, the contribution of saproxylic arthropods is still insufficiently quantified. They are considered to contribute between 10 to 20% to wood decomposition [27, 28]. Do saproxylic beetles in particular operate mainly as coarse decomposers of deadwood thereby facilitating wood decomposition by enlarging the surface or might they influence the efficiency of wood decomposition by acting as vectors of fungal spores as suggested by several studies [28]? Examples refer to the distribution of plant diseases e.g. [26] or the mutualistic relationships of ambrosia beetles that actively cultivate fungal gardens in trees [59,60]. Very little is known how phoretic fungi that are carried on the surfaces of beetle exoskeletons influence wood decomposition [61]. In a recent investigation [19] Strid et al. found indirect evidence that saproxylic beetles determine fungal community composition by vectoring spores. The authors had come to this conclusion after comparing fungal communities in spruce stems with spruce stems from which bark beetles had been experimentally excluded. We analysed our data tables for that kind of interrelationships. We used all beetles collected during one season and cross-correlated these with fungal MOTUs that were identified from the same deadwood stacks used to attract beetles by the FITs. We were able to identify some, namely 18, significant correlations (Fig 5) of which most were saproxylics or mycetophages. Although the three phytophagous species are unlikely to be taken into account as specific vectors they might play a passive role for spore transport.

None of the identified fungal-beetle species pairs have been described yet. However, most of the beetles are saproxylic and the fungal genera are known as partners of such associations [53,62].

One therefore might see the significant correlations as a basis to formulate hypothesis about yet the unrecognized interspecific connections. Furthermore, the identified correlations can be used to validate the current ecological characterisation of beetles. For example, both ambrosia beetles *Xyleborus saxeseni* and *X*. *domesticus* (Scolytidae) were positively associated with the early colonizing *Hypoxylon fragiforme* but negatively with the usually later arriving *H*. *rubiginosum* confirming the ecological classification of these species [63]. Correspondingly, *Denticollis linearis* (Cantharidae) and *Microscydmus minimus* (Scydmaenidae) are known to be associated with old beech deadwood and *Litargus connexus* (Mycetophagidae) lives under bark which is detaching from the stem. Even if there are alternative causes explaining these correlations like microclimate or substrate the results offer the opportunity to close gaps in our knowledge for example of larval biology which is often the basis for the functional assortment of species.

The lack of identifying such associations [64] indicates therefore the preliminary character of our study and makes a plea for more investigations. Understanding the interactions between saproxylic arthropods and wood-inhabiting fungi might contribute significantly to our understanding of the mutualistic relationship between wood fungi and beetles and the decomposition performance of deadwood by fungal assemblages.

## Supporting Information

S1 Fig Overview of three German Biodiversity Exploratories. In detail: maps of the “Swabian Alb”, Hainich-Dün and “Schorfheide-Chorin”, including the locations of the presented investigation plots.
(PDF)

S2 Fig Sampling Design. (A) Positions of the deadwood stacks and traps (left photographs) and number of samples collected from the canopy and ground stratum in the Swabian Alb, Hainich-Dün and Schorfheide-Chorin and different forest management types (acf = age-class forest, ext = extensively managed, unm = unmanaged). Beetles were collected by FITs installed in front of deadwood enrichments. Fungi were identified from drill samples extracted from larges sized canopy and ground deadwood logs (see text). (B) Sampling scheme visualized using Treemap v. 3.1.0. (Macrofocus, Zurich, Switzerland) in squarified layout. Items are hierarchically grouped by region (HEW = Hainich, AEW = Alb, SEW = Schorfheide) and stratum (canopy and ground). Treemap cells represent the single deadwood stack and the corresponding management type (grey = age-class forests, red = extensively managed forests and green = unmanaged forests).
(PDF)

S3 Fig Icicle-like adjaceny chart: made with in-cell bars representing the taxonomic distribution of the fungal sequences and MOTUs found as well as the plots where these MOTUs were found (heatmapped by number of sequences). (A) quantiles, sum of sequences in MOTU, MOTU name, MOTU reference sequence accession number, icicle chart based on abbreviated NCBI taxonomy path, ecological information, (B) in addition, clone origin of MOTU reference sequence,nearest BLASTn hit accession number, length of MOTU reference sequence, and distribution of MOTUs across samples.
(PDF)

S4 Fig Individual based rarefaction curves with confidence intervals computedfor all combined wood-inhabiting fungi (left) and beetles (right). Due to little species overlap between regions sampling was not representative. Beetle but not fungal richness differed significantly between regions.
(PDF)
