# Supplementary material for: Diversity and Interactions of Wood-Inhabiting Fungi and Beetles after Deadwood Enrichment
Source: PLoS One. 2015 Nov 24;10(11):e0143566. doi: 10.1371/journal.pone.0143566 (PMC4657976; doi:10.1371/journal.pone.0143566)
Supplement: S1 Fig — In detail: maps of the “Swabian Alb”, Hainich-Dün and “Schorfheide-Chorin”, including the locations of the presented investigation plots. (PDF) [file pone.0143566.s001.pdf]

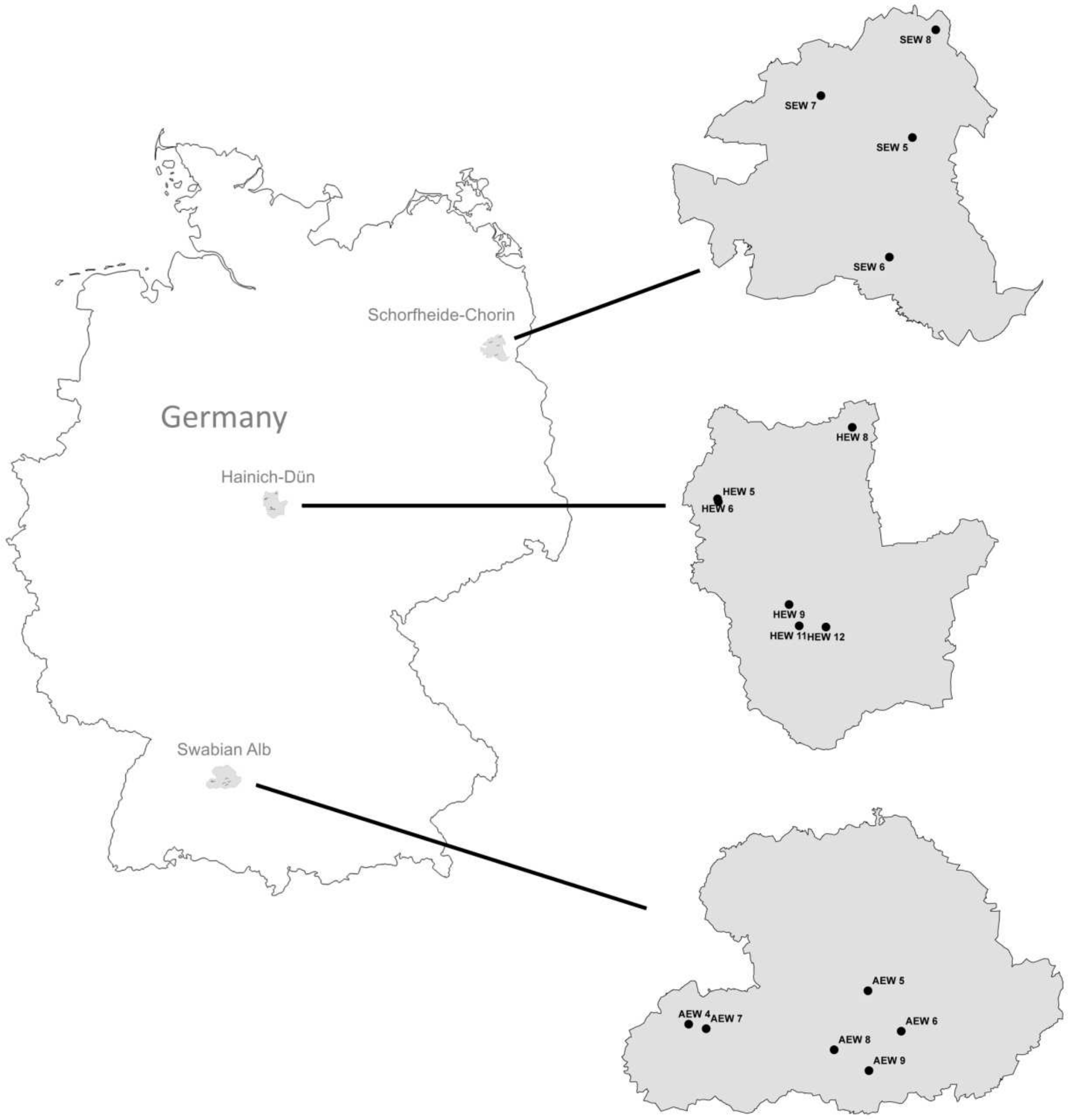

S1 Fig. Overview of three German Biodiversity Exploratories. In detail: maps of the “Swabian Alb”, Hainich-Dün and “Schorfheide-Chorin”, including the locations of the presented investigation plots.
