# Supplementary material for: Diversity and Interactions of Wood-Inhabiting Fungi and Beetles after Deadwood Enrichment
Source: PLoS One. 2015 Nov 24;10(11):e0143566. doi: 10.1371/journal.pone.0143566 (PMC4657976; doi:10.1371/journal.pone.0143566)
Supplement: S2 Fig — (A) Positions of the deadwood stacks and traps (left photographs) and number of samples collected from the canopy and ground stratum in the Swabian Alb, Hainich-Dün and Schorfheide-Chorin and different forest management types (acf = age-class forest, ext = extensively managed, unm = unmanaged). Beetles were collected by FITs installed in front of deadwood enrichments. Fungi were identified from drill samples extracted from larges sized canopy and ground deadwood logs (see text). (B) Sampling scheme visualized using Treemap v. 3.1.0. (Macrofocus, Zurich, Switzerland) in squarified layout. Items are hierarchically grouped by region (HEW = Hainich, AEW = Alb, SEW = Schorfheide) and stratum (canopy and ground). Treemap cells represent the single deadwood stack and the corresponding management type (grey = age-class forests, red = extensively managed forests and green = unmanaged forests). (PDF) [file pone.0143566.s002.pdf]

A

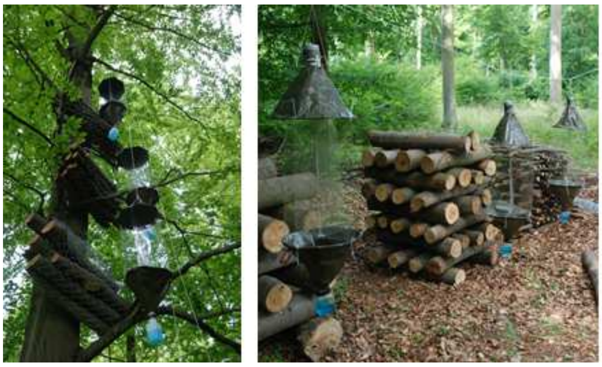

	Alb	Hainich	Chorin
Canopy	3	6	3
Ground	6	6	4
Acf	4	4	3
Ext	5	4	0
unm	0	4	4

B

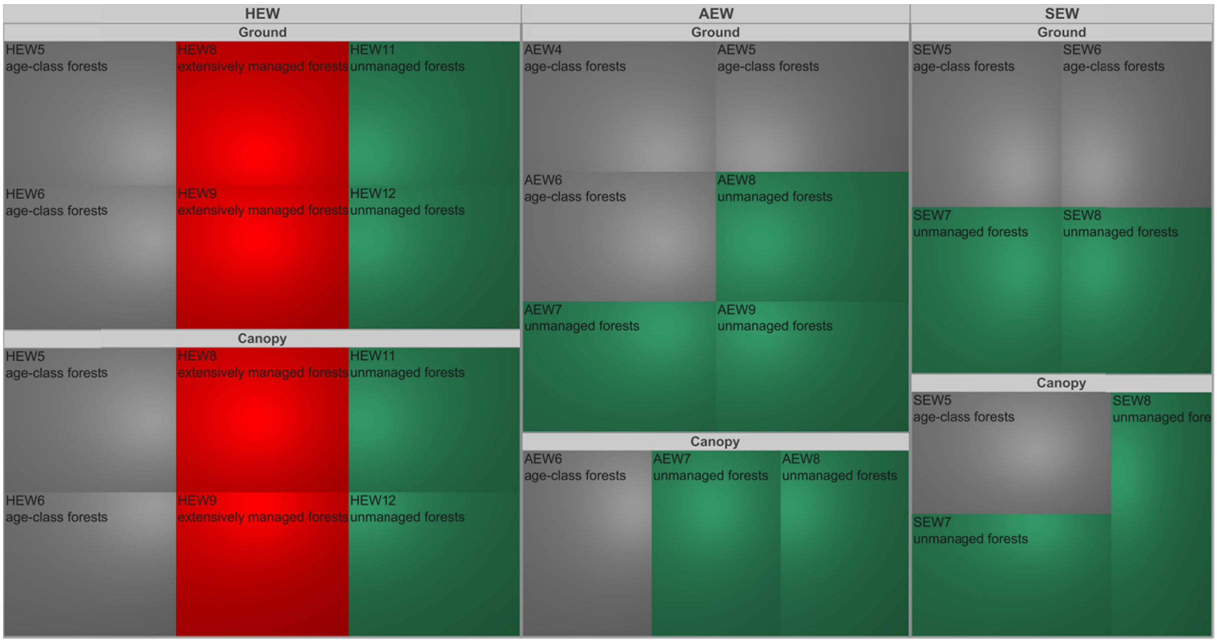

S2 Fig. Sampling Design. (A) Positions of the deadwood stacks and traps (left photographs) and number of samples collected from the canopy and ground stratum in the Swabian Alb, Hainich-Dün and Schorfheide-Chorin and different forest management types (acf=age-class forest, ext=extensively managed, unm=unmanaged). Beetles were collected by FITs installed in front of deadwood enrichments. Fungi were identified from drill samples extracted from larges sized canopy and ground deadwood logs (see text). (B) Sampling scheme visualized using Treemap v. 3.1.0. (Macrofocus, Zurich, Switzerland) in squarified layout. Items are hierarchically grouped by region (HEW = Hainich, AEW = Alb, SEW = Schorfheide) and stratum (canopy and ground). Treemap cells represent the single deadwood stack and the corresponding management type (grey = age-class forests, red = extensively managed forests and green = unmanaged forests).
