# Supplementary material for: Diversity and Interactions of Wood-Inhabiting Fungi and Beetles after Deadwood Enrichment
Source: PLoS One. 2015 Nov 24;10(11):e0143566. doi: 10.1371/journal.pone.0143566 (PMC4657976; doi:10.1371/journal.pone.0143566)
Supplement: S3 Fig — (A) quantiles, sum of sequences in MOTU, MOTU name, MOTU reference sequence accession number, icicle chart based on abbreviated NCBI taxonomy path, ecological information, (B) in addition, clone origin of MOTU reference sequence,nearest BLASTn hit accession number, length of MOTU reference sequence, and distribution of MOTUs across samples. (PDF) [file pone.0143566.s003.pdf]

S3 Fig. Icicle-like adjaceny chart: made with in-cell bars representing the taxonomic distribution of the fungal sequences and MOTUs found as well as the plots where these MOTUs were found (heatmapped by number of sequences). (A) quantiles, sum of sequences in MOTU, MOTU name, MOTU reference sequence accession number, icicle chart based on abbreviated NCBI taxonomy path, ecological information, (B) in addition, clone origin of MOTU reference sequence,nearest BLASTn hit accession number, length of MOTU reference sequence, and distribution of MOTUs across samples.

A

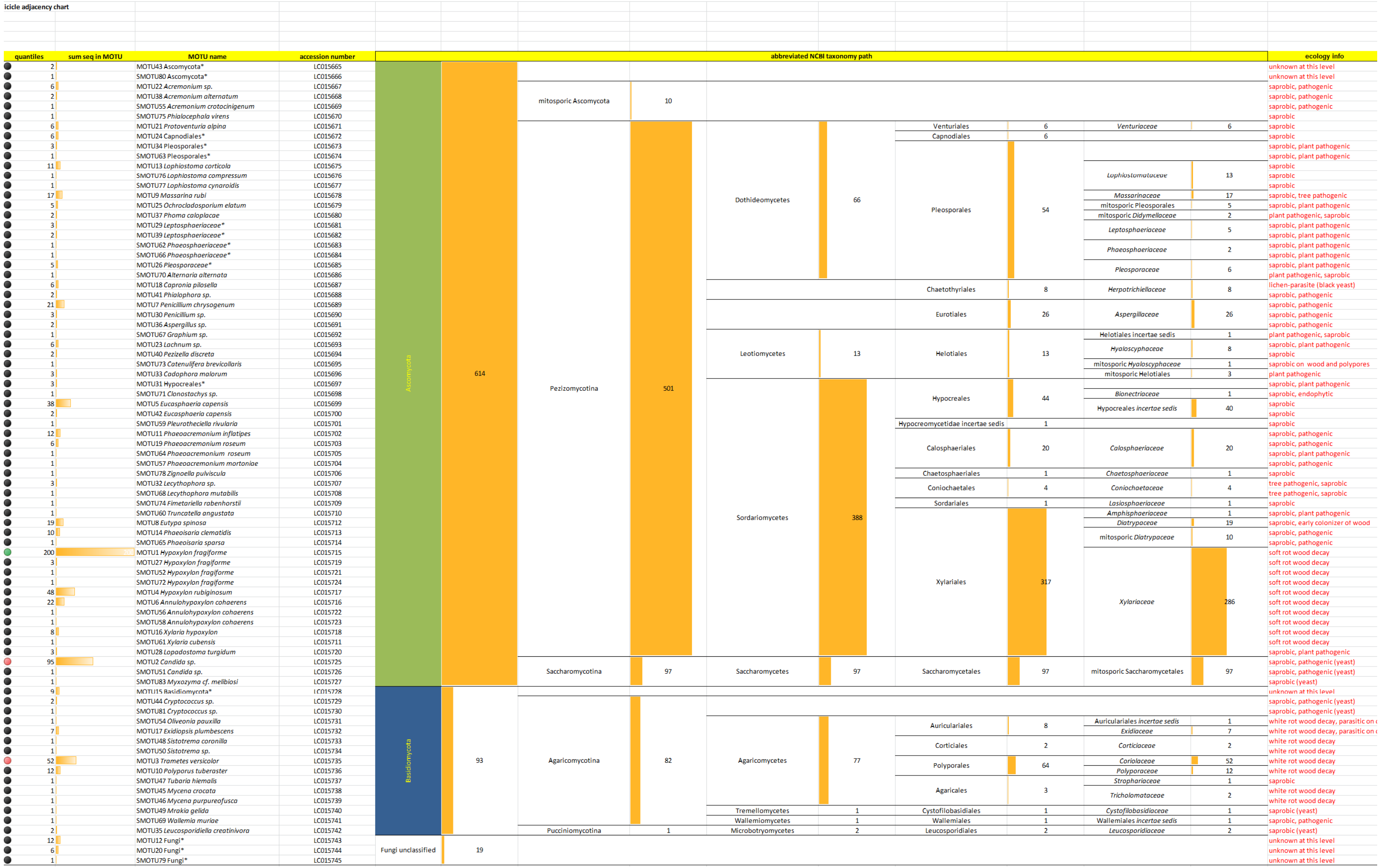

B

exploratory: SEW... Schorfheide-Chorin; HEW... Hainich-Dün; AEW... Schwäbische Alb

stratum code: ...C crown (K in clone name); ...G ground (B in clone name)

heatmap between 0 (white) and 26 (green)

MOTU name	clone (MOTU reference seq)	near accession	nt lenght of MOTU reference seq	MOTU reference seq	matrix
MOTU43 Ascomycota*	K02AEW7	AY568066	526 nt	AAGTCGTAACAAGGTTTCCGTAGGTGAACC	AEW7C 1, HEW9G 1
SMOTU80 Ascomycota*	K01SEW5	EF373561	523 nt	AAGTCGTAACAAGGTTTCCGTAGGTGAACC	SEW5C 1
MOTU22 *Acremonium sp.*	K26SEW7	HQ829107	583 nt	AAGTCGTAACAAGGTCTCCGTTGGTGAACC	SEW7G 3, SEW6G 2, SEW7C 1
MOTU38 *Acremonium alternatum*	K142HEW9	DQ914662	561 nt	AAGTCGTAACAAGGTCTCCGTTGGTGAACC	HEW9C 2
SMOTU55 *Acremonium crotocinigenum*	K143HEW9	GU934498	597 nt	AAGTCGTAACAAGGTCTCCGTTGGTGAACC	HEW9C 1
SMOTU75 *Phialocephala virens*	B30SEW5	AB671505.2	549 nt	AAGTCGTAACAAGGTTTCCGTAGGTGAACC	SEW5G 1
MOTU21 *Protoventuria alpina*	K085HEW6	EU035444	583 nt	AAGTCGTAACAAGGTTTCCGTAGGTGAACC	AEW6C 2, HEW5C 2, HEW6C 2
MOTU24 Capnodiales*	K10AEW6	JX270582	547 nt	AAGTCGTAACAAGGTCTCCGTAGGTGAACC	AEW6C 1, HEW9G 1, HEW9C 1, HEW11G 1, HEW12G 1, SEW6G 1
MOTU34 Pleosporales*	K14AEW6	HM116750	528 nt	AAGTCGTAACAAGGTTTCCGTAGGCGAACC	AEW6C 2, AEW6G 1
SMOTU63 Pleosporales*	B125HEW9	HQ631059	580 nt	AAGTCGTAACAAGGTTTCCGTAGGTGAACC	HEW9G 1
MOTU13 *Lophiostoma corticola*	K21AEW6	HM116751	537 nt	AAGTCGTAACAAGGTTTCCGTAGGTGAACC	HEW6G 4, HEW8C 3, AEW6C 2, HEW5C 1, HEW6C 1
SMOTU76 *Lophiostoma compressum*	B06AEW6	JN942963	544 nt	AAGTCGTAACAAGGTTTCCGTAGGTGAACC	AEW6G 1
SMOTU77 *Lophiostoma cynaroidis*	K30AEW6	HM123518	542 nt	AAGTCGTAACAAGGTTTCCGTAGGTGAACC	AEW6C 1
MOTU9 *Massarina rubi*	B27AEW6	AF383963	543 nt	AAGTCGTAACAAGGTTTCCGTAGGTGAACC	SEW5G 5, HEW5C 3, AEW6G 2, AEW8G 2, HEW9G 2, AEW9G 1, HEW8C 1, SEW6G 1
MOTU25 *Ochrocladosporium elatum*	B01SEW6	GU248334	541 nt	AAGTCGTAACAAGGTTTCCGTAGGTGAACC	SEW8C 3, SEW6G 1, SEW8G 1
MOTU37 *Phoma caloplacae*	K28AEW6	JQ238635	585 nt	AAGTCGTAACAAGGTTTCCGTAGGTGAACC	AEW6C 1, HEW8C 1
MOTU29 *Leptosphaeriaceae**	B22SEW6	HQ414598	589 nt	AAGTCGTAACAAGGTTTCCGTAGGTGAACC	HEW5C 2, SEW6G 1
MOTU39 *Leptosphaeriaceae**	K020HEW5n	DQ682586	548 nt	AAGTCGTAACAAGGTTTCCGTAGGTGAACC	HEW5C 2
SMOTU62 *Phaeosphaeriaceae**	K303HEW5	HQ324780	585 nt	AAGTCGTAACAAGGTTTCCGTAGGTGAACC	HEW5C 1
SMOTU66 *Phaeosphaeriaceae**	K39SEW8	JX978250	576 nt	AAGTCGTAACAAGGTTTCCGTAGGTGAGCC	SEW8C 1
MOTU26 *Pleosporaceae**	K42AEW6	JN120373	538 nt	AAGTCGTAACAAGGTTTCCGTAGGTGAACC	AEW6C 1, AEW7C 1, HEW9G 1, SEW5G 1, SEW7C 1
SMOTU70 *Alternaria alternata*	B01AEW5	KC253942	566 nt	AAGTCGTAACAAGGTCTCCGTAGGTGAACC	AEW5G 1
MOTU18 *Capronia pilosella*	B33AEW8	AF050256	624 nt	AAGTCGTAACAAGGTTTCCGTAGGTGAACC	AEW8G 3, HEW6G 2, SEW6G 1
MOTU41 *Phialophora sp.*	B19SEW5	FJ903315	544 nt	AAGTCGTAACAAGGTTTCCGTAGGTGAACC	HEW11C 1, SEW5G 1
MOTU7 *Penicillium chrysogenum*	B04HEW11	JN380201	581 nt	AAGTCGTAACAAGGTTTCCGTAGGTGAACC	HEW11G 8, HEW12G 5, HEW12C 5, HEW11C 3
MOTU30 *Penicillium sp.*	B12HEW11	HM222630	576 nt	AAGTCGTAACAAGGTTTCCGTAGGTGAACC	HEW11G 2, HEW12C 1
MOTU36 *Aspergillus sp.*	B42HEW12	AM901671	594 nt	AAGTCGTAACAAGGTTTCCGTAGGTGAACC	HEW12G 2
SMOTU67 *Graphium sp.*	B34HEW11	AB038426	575 nt	AAGTCGTAACAAGGTCTCCGTTGGTGAACC	HEW11G 1
MOTU23 *Lachnum sp.*	B13AEW9	KC007293	553 nt	AAGTCGTAACAAGGTTTCCGTAGGTGAACC	AEW9G 6
MOTU40 *Pezizella discreta*	B04AEW9	JF908571	547 nt	AAGTCGTAACAAGGTTTCCGTAGGTGAACC	AEW9G 2
SMOTU73 *Catenulifera brevicollaris*	B063HEW6	AB190385	556 nt	AAGTCGTAACAAGGTCTCCGTAGGTGAACC	HEW6G 1
MOTU33 *Cadophora malorum*	B39AEW9	DQ404350	547 nt	AAGTCGTAACAAGGTTTCCGTAGGTGAACC	AEW9G 1, SEW5G 1, SEW5C 1
MOTU31 Hypocreales*	K146HEW9	JQ647444	555 nt	AAGTCGTAACAAGGTCTCCGTTGGTGAACC	HEW9C 3
SMOTU71 *Clonostachys sp.*	B17HEW12	KC007312	561 nt	AAGTCGTAACAAGGTCTCCGTTGGTGAACC	HEW12G 1
MOTU5 *Eucasphaeria capensis*	B06SEW6	EU272516	574 nt	AAGTCGTAACAAGGTCTCCGTTGGTGAACC	HEW6G 11, HEW9C 6, HEW8G 5, SEW6G 5, HEW11G 3, HEW12G 3, HEW5G 2, HEW11C 1, HEW12C 1, SEW8G 1
MOTU42 *Eucasphaeria capensis*	B28SEW8	EF110619	529 nt	AAGTCGTAACAAGGTCTCCGTTGGTGAACC	SEW8G 2
SMOTU59 *Pleurotheciella rivularia*	B38SEW5	JQ429161	590 nt	AAGTCGTAACAAGGTCTCCGTTGGTGAACC	SEW5G 1
MOTU11 *Phaeoacremonium inflatipes*	K01AEW6	AF197990	595 nt	AAGTCGTAACAAGGTCTCCGTTGGTGAACC	AEW6C 6, AEW8G 3, HEW8C 1, HEW9G 1, SEW8G 1
MOTU19 *Phaeoacremonium roseum*	B01AEW8	EU128046	589 nt	AAGTCGTAACAAGGTCTCCGTTGGTGAACC	AEW8G 5, SEW5G 1
SMOTU64 *Phaeoacremonium roseum*	B26SEW5	HQ823751	578 nt	AAGTCGTAACAAGGTCTCCGTTGGTGAACC	SEW5G 1
SMOTU57 *Phaeoacremonium mortoniae*	B36AEW6	EU427312	591 nt	AAGTCGTAACAAGGTCTCCGTTGGTGAACC	AEW6G 1
SMOTU78 *Zignoella pulviscula*	B034HEW5n	AF178543	533 nt	AAGTCGTAACAAGGTCTCCGTTGGTGAACC	HEW5G 1
MOTU32 *Lecythophora sp.*	B22AEW9	HE998721	550 nt	AAGTCGTAACAAGGTTTCCGTAGGTGAACC	AEW9G 2, SEW8G 1
SMOTU68 *Lecythophora mutabilis*	B02SEW7	HE610334	574 nt	AAGTCGTAACAAGGTTTCCGTAGGTGAACC	SEW7G 1
SMOTU74 *Fimetariella rabenhorstii*	B11AEW9	FJ872072	554 nt	AAGTCGTAACAAGGTCTCCGTTGGTGAACC	AEW9G 1
SMOTU60 *Truncatella angustata*	B33AEW9	KC241879	588 nt	AAGTCGTAACAAGGTCTCCGTTGGTGAACC	AEW9G 1
MOTU8 *Eutypa spinosa*	B45AEW4	EF155486	625 nt	AAGTCGTAACAAGGTCTCCGTTGGTGAACC	AEW7G 18, AEW4G 1
MOTU14 *Phaeoisaria clematidis*	B28AEW6	EU552148	574 nt	AAGTCGTAACAAGGTCTCCGTTGGTGAACC	AEW8G 3, HEW5G 2, SEW6G 2, AEW6G 1, HEW9G 1, SEW8C 1
SMOTU65 *Phaeoisaria sparsa*	B12SEW6	EU552148	577 nt	AAGTCGTAACAAGGTCTCCGTTGGTGAACC	SEW6G 1
MOTU1 *Hypoxylon fragiforme*	K065HEW6	EF155528	625 nt	AAGTCGTAACAAGGTCTCCGTTGGTGAACC	SEW7C 24, SEW8G 19, SEW5C 18, SEW8C 18, SEW7G 17, HEW6C 16, HEW5G 15, HEW5C 10, AEW6C 9, SEW6G 8, AEW7C 7, HEW8G 7, HEW8C 7, HEW9G 6, HEW9C 6, SEW5G 4, HEW11C 3, HEW12G 3, AEW8G 1, HEW11G 1, HEW12C 1
MOTU27 *Hypoxylon fragiforme*	K04SEW5	EU686087	646 nt	AAGTCGTAACAAGGTCTCCGTTGGTGAACC	SEW5C 3
SMOTU52 *Hypoxylon fragiforme*	K47AEW6	FM200459	624 nt	AAGTCGTAACAAGGTCTCCGTTGGTGAACC	AEW6C 1
SMOTU72 *Hypoxylon fragiforme*	K241HEW5	EF155528	559 nt	AAGTCGTAACAAGGTTTCCGTAGGTGAACC	HEW5C 1
MOTU4 *Hypoxylon rubiginosum*	B26AEW8	EF026143	615 nt	AAGTCGTAACAAGGTCTCCGTTGGTGAACC	AEW6G 12, AEW4G 9, AEW8G 8, HEW6G 7, AEW5G 6, AEW9G 3, AEW8C 2, HEW6C 1
MOTU6 *Annulohypoxylon cohaerens*	B04SEW5	EF026140	591 nt	AAGTCGTAACAAGGTCTCCGTTGGTGAACC	AEW4G 14, HEW5G 4, SEW5G 2, AEW9G 1, HEW6G 1
SMOTU56 *Annulohypoxylon cohaerens*	K010HEW5n	EF026140	594 nt	AAGTCGTAACAAGGTCTCCGTTGGTGAACC	HEW5C 1
SMOTU58 *Annulohypoxylon cohaerens*	B31SEW8	EF026140	590 nt	AGGTCGTAACAAGGTCTCCGTTGGTGAACC	SEW8G 1
MOTU16 *Xylaria hypoxylon*	B02AEW6	GU300096	581 nt	AAGTCGTAACAAGGTCTCCGTTGGTGAACC	AEW6G 8
SMOTU61 *Xylaria cubensis*	B46SEW5	AF163032	587 nt	AAGTCGTAACAAGGTCTCCGTTGGTGAACC	SEW5G 1
MOTU28 *Lopadostoma turgidum*	K33SEW5	EU686107	600 nt	AAGTCGTAACAAGGTCTCCGTTGGTGAACC	SEW5C 3
MOTU2 *Candida sp.*	B42HEW11	HE998694	628 nt	AAGTCGTAACAAGGTTTCCGTAGGTGAACC	HEW11C 16, HEW8C 13, HEW12C 13, HEW8G 12, HEW9G 12, HEW12G 10, HEW11G 9, HEW9C 7, HEW5C 2, AEW7C 1
SMOTU51 *Candida sp.*	B48HEW12	HE998694	627 nt	AAGCCGTAACAAGGTTTCCGTAGGTGAACC	HEW12G 1
SMOTU83 *Myxozyma cf. melibiosi*	K18SEW8	AF530607	431 nt	AAGTCGTAACAAGGTTTCCGTAGGTGAACC	SEW8C 1
MOTU15 Basidiomycota*	B01AEW4	AJ279471	622 nt	AAGTCGTAACAAGGTTTCCGTAGGTGAACC	AEW7G 8, AEW4G 1
MOTU44 *Cryptococcus sp.*	B005HEW5	HQ875392	522 nt	AAGTCATAACAAGGTTTCCGTAGGTGAACC	HEW5G 2
SMOTU81 *Cryptococcus sp.*	K15SEW8	AY206426	516 nt	AAGTCATAACAAGGTTTCCGTAGGTGAACC	SEW8C 1
SMOTU54 *Oliveonia pauxilla*	B139HEW8n	HQ441577	600 nt	AAGTCGTAACAAGGTTTCCGTAGGTGAACC	HEW8G 1
MOTU17 *Exidiopsis plumbescens*	B02AEW9	AF395309	591 nt	AAGTCGTAACAAGGTTTCCGTAGGTGAACC	AEW9G 7
SMOTU48 *Sistotrema coronilla*	B14SEW5	DQ397337	652 nt	AAGTCGTAACAAGGTTTCCGTAGGTGAACC	SEW5G 1
SMOTU50 *Sistotrema sp.*	B08AEW8	FJ609284	631 nt	AAGTCGTAACAAGGTTTCCGTAGGTGAACC	AEW8G 1
MOTU3 *Trametes versicolor*	B03AEW5	JQ673022	619 nt	AAGTCGTAACAAGGTTTCCGTAGGTGAACC	AEW8C 24, AEW5G 19, HEW6C 5, AEW7C 4
MOTU10 *Polyporus tuberaster*	K01AEW7	AF516593	652 nt	AAGTCGTAACAAGGTTTCCGTAGGTGAACC	AEW7C 12
SMOTU47 *Tubaria hiemalis*	K072HEW6	FJ717494	689 nt	AAGTCGTAACAAGGTTTCCGTAGGTGAACC	HEW6C 1
SMOTU45 *Mycena crocata*	B35SEW5	JF908492	719 nt	AAGTCGTAACAAGGTTTCCGTAGGTGAACC	SEW5G 1
SMOTU46 *Mycena purpureofusca*	B06AEW9	HQ604765	695 nt	AAGTCGTAACAAGGTTTCCGTAGGTGAACC	AEW9G 1
SMOTU49 *Mrakia gelida*	B141HEW8n	AF144485	637 nt	AAGTCGTAACAAGGTTTCCGTAGGTGAACC	HEW8G 1
SMOTU69 *Wallemia muriae*	K32HEW11	AY302534	567 nt	AAGTCGTAACAAGGTTTCCGTAGGTGAACC	HEW11C 1
MOTU35 *Leucosporidiella creatinivora*	B33HEW11	AF444629	604 nt	AAGTCGTAACAAGGTTTCCGTAGGTGAACC	HEW11G 1, HEW12C 1
MOTU12 Fungi*	B20SEW5	DQ068345	590 nt	AAGTCGTAACAAGGTCTCCGTTGGTGAACC	SEW7G 5, HEW12C 4, SEW5G 2, HEW11C 1
MOTU20 Fungi*	B11SEW5	JX978250	587 nt	AAGTCGTAACAAGGTTTCCGTAGGTGAACC	SEW6G 3, SEW5G 2, HEW5C 1
SMOTU79 Fungi*	K26SEW8	JX978239	528 nt	AAGTCGTAACAAGGTTTCCGTAGGTGAACC	SEW8C 1
