# Supplementary material for: Diversity and Interactions of Wood-Inhabiting Fungi and Beetles after Deadwood Enrichment
Source: PLoS One. 2015 Nov 24;10(11):e0143566. doi: 10.1371/journal.pone.0143566 (PMC4657976; doi:10.1371/journal.pone.0143566)
Supplement: S4 Fig — Due to little species overlap between regions sampling was not representative. Beetle but not fungal richness differed significantly between regions. (PDF) [file pone.0143566.s004.pdf]

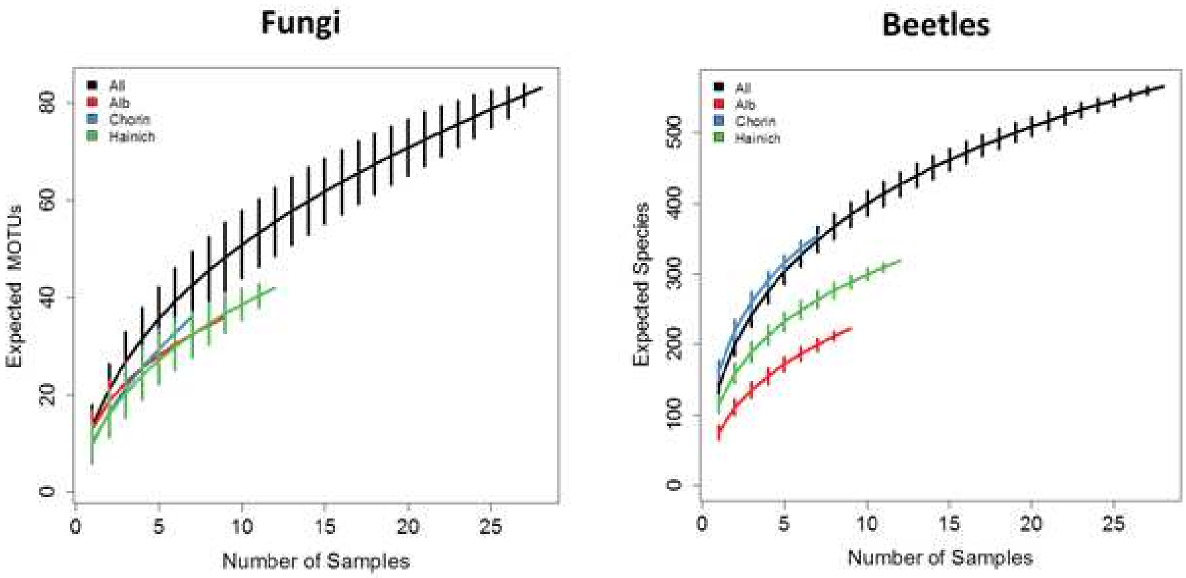

S4 Fig. Individual based rarefaction curves with confidence intervals computedfor all combined wood-inhabiting fungi (left) and beetles (right). Due to little species overlap between regions sampling was not representative. Beetle but not fungal richness differed significantly between regions.
